# KRAS G12C inhibitor combination therapies: current evidence and challenge

**DOI:** 10.3389/fonc.2024.1380584

**Published:** 2024-05-02

**Authors:** Hirotaka Miyashita, Shumei Kato, David S. Hong

**Affiliations:** ^1^ Hematology and Oncology, Dartmouth Cancer Center, Lebanon, NH, United States; ^2^ Center for Personalized Cancer Therapy and Division of Hematology and Oncology, Department of Medicine, University of California San Diego Moores Cancer Center, La Jolla, CA, United States; ^3^ Department of Investigational Cancer Therapeutics, Division of Cancer Medicine, The University of Texas MD Anderson Cancer Center, Houston, TX, United States

**Keywords:** KRAS, KRAS G12C inhibitors, combination (combined) therapy, MAPK, RTK inhibitors, mTOR, immunotherapy

## Abstract

Although KRAS G12C inhibitors have proven that KRAS is a “druggable” target of cancer, KRAS G12C inhibitor monotherapies have demonstrated limited clinical efficacy due to primary and acquired resistance mechanisms. Multiple combinations of KRAS G12C inhibitors with other targeted therapies, such as RTK, SHP2, and MEK inhibitors, have been investigated in clinical trials to overcome the resistance. They have demonstrated promising efficacy especially by combining KRAS G12C and EGFR inhibitors for KRAS G12C-mutated colorectal cancer. Many clinical trials of combinations of KRAS G12C inhibitors with other targeted therapies, such as SOS1, ERK, CDK4/6, and wild-type RAS, are ongoing. Furthermore, preclinical data have suggested additional promising KRAS G12C combinations with YAP/TAZ-TEAD inhibitors, FAK inhibitors, and farnesyltransferase inhibitors. The combinations of KRAS G12C inhibitors with immunotherapies and chemotherapies have also been investigated, and the preliminary results were reported. More recently, KRAS-targeted therapies not limited to KRAS G12C are being developed, potentially broadening the treatment landscape of KRAS-mutated cancers. Rationally combining KRAS inhibitors with other therapeutics is likely to play a significant role in future treatment for KRAS-mutated solid tumors.

## Introduction

Since the discovery of RAS oncogenes in the 1960s (1), concerted efforts have been made to understand the epidemiology, molecular biology, and how to target this gene’s mutation as a cancer treatment. Comprehensive genomic analyses have demonstrated that KRAS, one of the three major RAS oncogenes, is frequently altered in multiple types of cancer, including colorectal cancer (CRC) (2), non-small cell lung cancer (NSCLC) (3), and pancreatic ductal adenocarcinoma (PDAC) (4). Because of the high prevalence of the alteration, KRAS has been an attractive treatment target for cancer. The complex signaling cascades of RAS, including the most pivotal RAS–RAF–MEK–ERK pathway (MAPK pathway), have been gradually identified, which has justified the approach to target altered RAS as a cancer treatment as well (5).

However, unlike the treatments targeting other gene alterations, such as EGFR mutations in NSCLC (6), targeting KRAS has yet to be successful enough to revolutionize KRAS-mutated cancer treatment. The biggest challenge in targeting altered KRAS was its inaccessible binding surface and high affinity to guanosine triphosphate (GTP) and guanosine diphosphate (GDP) (7). It took a long time for a new technology to allow the discovery of a small molecule that binds to a specific KRAS G12C mutation (8). The small molecules have shown anti-tumor efficacy and reasonable safety in preclinical models and clinical trials for NSCLC (9, 10). Although KRAS G12C mutation is not the most common alteration of KRAS, the success of KRAS G12C inhibitors has proven the feasibility of targeting KRAS, which has led to further investigations. As of the time this review was written, two KRAS G12C inhibitors have been approved by the Food and Drug Administration (FDA) as a subsequent treatment for KRAS G12C-mutated NSCLC (11). KRAS G12C inhibitor monotherapies have not demonstrated convincing efficacy for other indications, such as CRC or PDAC. Therefore, combining KRAS G12C inhibitors with other cancer treatments has been studied, and we see more and more evidence recently. This review article summarizes the latest evidence from the clinical trials of KRAS G12C inhibitor combinations and discusses other possible combinations with their preclinical evidence.

## Overview of KRAS G12C inhibitors and other KRAS inhibitors

As of December 2023, two KRAS G12C inhibitors (sotorasib and adagrasib) have been approved by the FDA. Sotorasib was granted accelerated approval from the FDA in May 2021 for KRAS G12C-mutated advanced NSCLC that has progressed on at least one systemic treatment based on the initial result of the CodeBreak 100 trial (10). The CodeBreak 100 was a single-arm, phase II trial of sotorasib monotherapy in patients with KRAS G12C-mutated advanced NSCLC previously treated with standard therapies. It showed an objective response rate (ORR) of 37.1% with median progression-free survival (mPFS) and median overall survival (mOS) of 6.8 and 12.5 months, respectively. In December 2022, the FDA granted accelerated approval to the second KRAS G12C inhibitor, adagrasib, for previously treated, advanced KRAS G12C-mutated NSCLC. This was based on the result of the KRYSTAL-1 trial, a single arm, phase I/II trial for pretreated advanced NSCLC with KRAS G12C mutation, which demonstrated an ORR of 42.9% with mPFS and mOS of 6.5 and 12.6 months, respectively (12). Both sotorasib and adagrasib are listed as recommended subsequent therapy for KRAS G12C-mutated NSCLC in the NCCN guidelines for NSCLC (11). There are multiple other KRAS G12C inhibitors under investigation in early phase clinical trials, including LY3537982 (13), JDQ443 (14), and divarasib (15). These molecules, including sotorasib and adagrasib, are called KRAS G12C (OFF) inhibitors, which bind to GDP-bound KRAS G12C to prevent the activation of the inactive KRAS G12C. On the other hand, RMC-6291 is a RAS (ON) inhibitor, which binds to a chaperone protein cyclophilin A to form an inhibitory complex and inhibit downstream activation of GTP-bound KRAS. In a phase I clinical trial, RMC-6291 showed anti-tumoral efficacy in patients with KRAS G12C-mutated solid tumors even with previous KRAS G12C (OFF) inhibitor use (16). Furthermore, FMC-376, a novel dual KRAS G12C (ON) and (OFF) inhibitor, has shown anti-tumor activity in vivo, suggesting the ability to overcome the resistance to KRAS G12C (OFF) inhibitors (17).

Except for subsequent line treatment for KRAS G12C-mutated NSCLC, none of KRAS G12C inhibitors has been approved by the FDA, though previous clinical trials included different types of cancer. For example, the CodeBreak 100 enrolled 62 cases of advanced KRAS G12C-mutated CRC, and the ORR was 9.7% (18). It also included 38 cases of previously treated metastatic PDAC; the ORR was 21% with an mPFS of 4.0 months (19). The KRYSTAL-1 also investigated adagrasib monotherapy for previously treated advanced KRAS G12C-mutated CRC and showed an ORR of 19% with an mPFS of 5.6 months (20). Adagrasib monotherapy showed an ORR of 33.3% in pretreated advanced KRAS G12C-mutated PDAC (21).

## Current understanding of resistance mechanism to KRAS G12C inhibitors

As the previous clinical trials demonstrated, despite theoretically promising, KRAS G12C inhibitor monotherapy has yet to demonstrate an efficacy robust enough to change the treatment approach of KRAS G12C-mutated malignancies. Numerous resistance mechanisms to KRAS G12C inhibition have been revealed, with the two main categories being primary and acquired resistance.

The primary resistance to KRAS G12C inhibition is accountable for the insufficient response with KRAS G12C inhibitor monotherapies especially in CRC (18, 20). One of the critical resistance mechanisms is the presence of collateral (bypass) signaling. Although RAS is a driver of the RTK–RAS–MAPK axis, its upstream [receptor tyrosine kinase (RTK)] and downstream [mitogen-activated protein kinase (MAPK)] also get feedback from other pathways. This feedback reactivation contributes to the primary resistance to KRAS G12C inhibition (22). Other molecules that promote cell survival and proliferation with KRAS G12C inhibition include PI3K, AKT, mTOR, SHP2, SOS1, and CDK4 (23). In addition to the collateral signaling effect, genomic co-alterations play a role in the primary resistance. For NSCLC, KEAP1, SMARCA4, and CDKN2A co-alterations were significant determinants of inferior clinical outcomes with KRAS G12C inhibition (24). Additional baseline RAS alterations, including amplification, are possibly associated with poor response to KRAS G12C inhibitors (24).

On the other hand, acquired resistance leads to treatment refractoriness in the tumors that initially respond to KRAS G12C inhibitors. The primary mechanism of acquired resistance is the development of new genomic alterations during treatment. Acquired alterations can happen both in KRAS and other genes. For example, among 38 patients with KRAS G12C mutated solid tumors in KRYSTAL-1, who progressed during adagrasib monotherapy, 17 patients demonstrated putative mechanisms of resistance to adagrasib, including new KRAS G12D/R/V/W, G13D, Q61H, R68S, H95D/Q/R, and Y96C mutations and amplification (25). The most common alteration was KRAS G12D mutation (four patients) followed by KRAS G13D mutation (three patients). Acquired alterations in other genes included MET amplification, activating mutations in NRAS, BRAF, MAP2K1, and RET, and oncogenic fusions of ALK, RET, BRAF, RAF1, and FGFR3, and loss of function mutation in NF1 and PTEN. At the time of acquired resistance, the original KRAS G12C mutation was identified in all 10 patients with tissue available for analysis. In the other 28 patients, only circulating tumor DNA was analyzed at the time of acquired resistance, and 22 patients demonstrated the original KRAS G12C mutation. Acquired KRAS alterations, RTK/RAS/MAPK/PI3K alterations, and acquired gene fusions appeared to be more common in CRC than in NSCLC in this cohort suggesting possible underlying differences in genomic instability or DNA damage-response mechanisms between CRC and NSCLC. Histologic transformation from adenocarcinoma to squamous cell carcinoma was also observed without other resistance mechanisms (25).

Rationally combining other agents with KRAS G12C inhibition is a possible solution for the primary resistance to KRAS G12C inhibition from collateral signaling. This is also inferred from the experience of enhanced efficacy from BRAF inhibition by the combination with EGFR inhibition in advanced BRAF V600E-mutated CRC (26). Due to the resistance from primary or acquired genomic co-alterations, combining therapies to cover multiple oncogenic targets may also enhance treatment efficacy.

As the results of the clinical trials on KRAS G12C inhibitor monotherapy have come out, multiple clinical trials of combining KRAS G12C inhibitor and other agents have been proposed and initiated.

## Currently available data on combining KRAS G12C inhibitors and other targeted therapies

As of January 2024, results from several clinical trials combining KRAS G12C inhibitors and other agents have been reported. The targets of the combined agents include RTKs (e.g., EGFR and HER2), SHP2, and MEK (**Table 1**).

**Table 1:** Summary of currently available data on combining KRAS G12C inhibitors with targeted therapies.

Target	Combination and NCT number	Phase	Population*	N	Toxicity data	Efficacy data	Reference
RTK	Cetuximab + adagrasib NCT03785249 (KRYSTAL-1)	1/2	Pretreated metastatic CRC with no prior KRAS G12C treatment	32	Common TRAE: nausea, diarrhea, vomiting G3-4 TRAE: 16% TRAE to d/c regimen: 0% for adagrasib, 16% for cetuximab	RR: 46% mPFS: 6.9m	(20)
	Cetuximab + divarasib NCT04449874	1b	Advanced or metastatic CRC (does not specify prior KRAS inhibition)	29	Common TRAE: rash, diarrhea, nausea G3-4 TRAE: 45% TRAE to d/c regimen: 0% for divarasib, 3.4% for cetuximab (rash)	Previous G12C inhibition (N = 5) RR: 60% G12C inhibition naïve (N = 24) RR: 62.5% mPFS: 8.1m	(27)
	Cetuximab + D1553 NCT04585035	2	Metastatic CRC with no prior KRAS G12C treatment	40	Common TRAE: rash, increased AST/ALT, paronychia G3-4 TRAE: 12.5% TRAE to d/c regimen: 2.5% (cetuximab related)	RR: 45.0% (not all confirmed) mPFS: 7.6m	(28)
	Panitumumab + sotorasib NCT05198934 (CodeBreaK 300)	3	Pretreated metastatic CRC	106	960 mg sotorasib cohort (N = 53) Common TRAE: hypomagnesemia, rash, dermatitis acneiform G3-4 TRAE: 35.8% TRAE to d/c regimen: 3.8% 240 mg sotorasib cohort (N = 53) Common TRAE: hypomagnesemia, rash, dermatitis acneiform G3-4 TRAE: 30.2% TRAE to d/c regimen: 1.9%	960 mg sotorasib cohort (N = 53) RR: 26.4% mPFS: 5.6m 240 mg sotorasib cohort (N = 53) RR: 5.7% mPFS: 3.9m	(29)
	Panitumumab + sotorasib NCT04185883 (CodeBreaK 101)	1b	Pretreated metastatic CRC	48	G3-4 TRAE: 27% (most commonly dermatologic) TRAE to d/c regimen: 0%	In the dose expansion cohort (N = 40) RR: 30% mPFS:5.7m mOS: 15.2m	(30)
	Panitumumab + FOLFIRI + sotorasib NCT04185883 (CodeBreaK 101)	1b	Pretreated metastatic CRC	46	Common TRAE: dermatitis acneiform, dry skin, nausea, and stomatitis G3-4 TRAE: 43% (most commonly dermatologic) TRAE to d/c regimen: 2% for sotorasib (ALT increased), 4% for panitumumab and 24% for FOLFIRI	RR: 55%	(31)
	Afatinib + sotorasib NCT04185883 (CodeBreaK 101)	1b	Pretreated advanced NSCLC	33	Common TRAE: diarrhea, nausea, vomiting G3-4 TRAE: 30% (most commonly diarrhea) TRAE to d/c regimen: 24% (most commonly diarrhea)	Cohort 1 (N = 10)† RR: 0% (Previous G12C inhibition, N = 4) RR: 33% (G12C inhibition naïve, N = 6) Cohort 2 (N = 23)† RR: 34.8%	(32)
SHP2	TNO155 + JDQ443 NCT04699188 (KontRASt-01)	1b	Pretreated advanced solid tumor	50‡	Common TRAE: peripheral edema, neutropenia, thrombocytopenia G3-4 TRAE: 36% (most commonly neutropenia) TRAE to d/c regimen: unavailable	Previous G12C inhibition RR (NSCLC, N = 12): 33% G12C inhibition naïve RR (NSCLC, N = 12): 33%	(33)
	RMC4630 + sotorasib NCT04185883 (CodeBreaK 101)	1b	Pretreated advanced solid tumor	27§	Common TRAE: edema, diarrhea, fatigue, dry mouth G3-4 TRAE: 22% (most commonly diarrhea) TRAE to d/c regimen: 11% (diarrhea, ascites, and AST increased)	Previous G12C inhibition RR (NSCLC, N = 5): 0% G12C inhibition naïve RR (NSCLC, N = 6): 50% One patient with ovarian cancer had PR	(34)
MEK	Trametinib + sotorasib NCT04185883 (CodeBreaK 101)	1b	Pretreated advanced solid tumor	41#	Common TRAE: diarrhea, rash, nausea G3-4 TRAE: 34.1% TRAE to d/c sotorasib: 4.9%	Previous G12C inhibition RR (CRC, N = 6): 16.7% RR (NSCLC, N = 3): 0% G12C inhibition naïve RR (CRC, N = 12): 8.3% RR (NSCLC, N = 15): 20%	(35)
	Avutometinib + sotorasib NCT05074810 (RAMP203)	1b/2	Pretreated advanced NSCLC	15	Common TRAEs: nausea, AST increase, diarrhea, fatigue, and pruritus G3-4 TRAE: ALP increase (20%), diarrhea (13%), pruritus (13%) The majority of TRAEs were grades 1–2	Previous G12C inhibition (N = 7) RR: 14.3% G12C inhibition naïve (N = 5) RR: 40%	(36)

ALP, alkaline phosphatase; AST, aspartate aminotransferase; CRC, colorectal cancer; d/c, discontinue; G3-4, grades 3 to 4; mPFS, median progression-free survival (months); N, number of participants; NSCLC, non-small cell lung cancer; PR, partial response; RR, response rate; RTK, receptor tyrosine kinase; SD, stable disease; TRAE, treatment-related adverse event.

*All participants should have KRAS G12C mutation.

†In cohort 1, 10 patients were given afatinib 20 mg and sotorasib 960 mg. In cohort 2, 23 patients were given afatinib 30 mg and sotorasib 960 mg.

‡24 patients with NSCLC, 19 patients with CRC, 3 patients with pancreatic cancer, 2 patients with biliary tract cancer, 1 patient with duodenal cancer, and 1 patient with ovarian cancer.

§11 patients with NSCLC, 9 with CRC, and 7 with other solid tumors.

#18 patients with NSCLC, 18 with CRC, and 5 with other solid tumors.

### Combination with an RTK-targeted therapy

One of the most critical mechanisms of primary resistance to KRAS G12C inhibition in CRC is the activation of EGFR signaling. Amodio et al. demonstrated that KRAS G12C-mutated CRC cell lines have high basal RTK activation, and KRAS G12C inhibition induces high phospho-ERK rebound. They also revealed the high efficacy of combining EGFR and KRAS G12C-targeted therapy in CRC cells, patient-derived organoids, and xenografts (22). This finding has led to multiple clinical trials to combine KRAS G12C inhibitors with RTK-targeted therapies predominantly for KRAS G12C-mutated CRC.

Cetuximab is a recombinant chimeric human/mouse IgG1 monoclonal antibody that inhibits EGFR and is approved for CRC and head and neck cancer. In KRYSTAL-1 (NCT03785249), the combination of cetuximab and adagrasib was given to 32 patients with previously treated metastatic KRAS G12C mutated CRC with no prior treatment with KRAS G12C inhibitors. The most common treatment-related adverse events (TRAEs) were nausea, diarrhea, and vomiting, and grade 3–4 TRAEs happened in 16% of the patients. No TRAE led to adagrasib discontinuation, but 16% of the patients discontinued cetuximab due to infusion-related reactions, malaise, and vascular flushing. The ORR was 46% with a median duration of response (mDoR) of 7.6 months and a mPFS of 6.9 months (20).

Divarasib is another covalent KRAS G12C inhibitor, combined with cetuximab in a phase Ib trial (NCT04449874). A total of 29 patients with advanced or metastatic KRAS G12C-mutated CRC were given cetuximab and divarasib. The common TRAEs were rash, diarrhea, and nausea. The grades 3–4 TRAEs were observed in 45%, with rash, diarrhea, and hypomagnesemia being the most common. One patient needed to discontinue cetuximab for rash, but no other TRAE leading to discontinuation of the regimen was observed. Among the 24 patients who had not previously received a KRAS G12C inhibitor, the ORR was 62.5% with a mDoR of 6.9 months. Their mPFS was 8.1 months. Among the five patients with previous KRAS G12C inhibitor exposure, three patients had a partial response (PR), and two had stable disease (SD) as their best response (27).

Cetuximab was also combined with D1553, another KRAS G12C inhibitor, in a phase II trial including 40 patients with metastatic KRAS G12C-mutated CRC with no previous KRAS G12C inhibition (NCT04585035). Among the 40 patients, the most common TRAEs were rash, increased AST/ALT, and paronychia. Grades 3–4 TRAEs were observed in 12.5% with rash being the most common. There was one case with TRAEs that required treatment discontinuation, but it was related to cetuximab. ORR was 45.0%, and mPFS was 7.6 months (28).

Panitumumab is a fully human monoclonal antibody that inhibits EGFR, which was combined with sotorasib for chemotherapy-refractory metastatic colorectal cancer with KRAS G12C mutation in CodeBreaK 300, a phase 3, multicenter, open-label, randomized trial (29). In the trial, 160 patients were enrolled and randomly assigned to receive sotorasib at a dose of 960 mg once daily and panitumumab, sotorasib 240 mg daily with panitumumab, or the investigator’s choice of trifluridine–tipiracil or regorafenib (standard care). The common TRAEs among the patients who received sotorasib and panitumumab combinations were hypomagnesemia, rash, and dermatitis acneiform. Among the 53 patients who received sotorasib 960 mg daily with panitumumab, 35.8% had grades 3–4 TRAEs, while 30.2% of the patients on the sotorasib 240 mg daily with panitumumab had grades 3–4 TRAEs. In each cohort, TRAEs leading to discontinuing the regimen were observed in 3.8% and 1.9%. As for the efficacy, ORR was 26.4% and 5.7%, and mPFR was 5.6 and 3.9 months in the sotorasib 960 mg plus panitumumab and sotorasib 240 mg plus panitumumab cohort, respectively. The hazard ratio for PFS in the 960-mg sotorasib plus panitumumab group compared to the standard care group was 0.49 (95% confidence interval: 0.30 to 0.80).

Panitumumab in combination with sotorasib was also investigated in a sub-cohort of CodeBreaK 101, a phase Ib trial (NCT04185883). This trial included patients with previously treated metastatic KRAS G12C-mutated CRC, and they were treated with a combination of panitumumab plus sotorasib with or without FOLFIRI (5-fluorouracil, leucovorin, and irinotecan). Among the 48 patients treated with panitumumab plus sotorasib, grades 3–4 TRAEs were observed in 27%, and dermatologic events were the most common. No TRAE leading to treatment discontinuation was observed. In the dose expansion cohort (N = 40), the ORR was 30% with mPFS and mOS of 5.7 and 15.2 months, respectively (30). Other 46 patients were treated with a combination of panitumumab, sotorasib, and FOLFIRI. The common TRAEs were dermatitis acneiform, dry skin, nausea, and stomatitis. Grades 3–4 TRAEs happened in 43% of the patients, with dermatologic events being the most common, and one patient needed to discontinue sotorasib due to increased ALT. The ORR was 55% with a mDoR of 7.0 months (31).

Afatinib is an inhibitor of multiple RTKs, including EGFR and HER2, used to treat NSCLC with specific EGFR mutations. In a sub-cohort of CodeBreaK 101 (NCT04185883), afatinib plus sotorasib was given to 33 patients with previously treated advanced KRAS G12C-mutated NSCLC. Diarrhea, nausea, and vomiting were the most common TRAEs, and 30% of the patients experienced grades 3–4 TRAEs, with diarrhea the most common. A total of 24% of the patients discontinued the treatment, most commonly due to diarrhea. In cohort 1 (afatinib 20 mg daily with sotorasib 960 mg daily), objective response was seen only in KRAS G12C inhibitor-naïve patients (33%, two out of six patients). In cohort 2 (afatinib 30 mg daily with sotorasib 960 mg daily), the ORR was 34.8% (32).

### Combination with a SHP2-targeted therapy

Due to the preclinical evidence that co-inhibition of SHP2 enhances the effectiveness of KRAS G12C inhibition (37), multiple clinical trials of combining an SHP2 inhibitor with a KRAS G12C inhibitor are ongoing. KontRASt-01 (NCT04699188) is a phase Ib trial that investigates the combination of TNO155 (a SHP2 inhibitor) and JDQ443 (a KRAS G12C inhibitor). Fifty patients with previously treated advanced KRAS G12C-mutated solid tumors (24 with NSCLC, 19 with CRC, and 7 with others) were given six different doses of TNO155 and JDQ443. Peripheral edema, neutropenia, and thrombocytopenia were the most common TRAEs, and grades 3–4 TRAEs happened in 36% of the patients, with neutropenia being the most common. Of the 12 patients with NSCLC who were naïve to KRAS G12C inhibition, 33% showed confirmed objective responses. Among 12 patients with NSCLC with a prior KRAS G12C inhibitor treatment, 33% had confirmed responses as well (33).

CodeBreaK 101 also has a sub-cohort to investigate the combination of RMC4630 (a SHP2 inhibitor) and sotorasib (NCT04185883). In this sub-cohort, 27 patients with previously treated advanced KRAS G12C-mutated solid tumors (11 with NSCLC, 9 with CRC, and 7 with others) were treated with RMC4630 and sotorasib. Edema, diarrhea, fatigue, and dry mouth were the most common TRAEs, and 22% experienced grades 3–4 TRAEs. The most common grades 3–4 TRAE was diarrhea, and 11% discontinued the treatment due to diarrhea, ascites, and AST increase. No response was observed in patients with NSCLC with previous KRAS G12C inhibition, but the ORR was 50% in KRAS G12C inhibition-naïve NSCLC patients. One patient with advanced ovarian cancer had a PR with an 81% reduction in tumor burden (34).

### Combination with an MEK-targeted therapy

Because of the preclinical evidence of the synergistic effect of an MEK inhibitor with a KRAS G12C inhibitor, clinical trials are investigating this combination. In a sub-cohort of CodeBreaK 101 (NCT04185883), trametinib in combination with sotorasib was given to 41 patients with previously treated advanced KRAS G12C-mutated solid tumors (18 with NSCLC, 18 with CRC, and 5 with others). The most common TRAEs were diarrhea, rash, and nausea. Of the participants, 34.1% experienced grades 3–4 TRAEs, and 4.9% of the patients had to discontinue the regimen due to TRAEs, most commonly diarrhea. Among the patients with previous KRAS G12C inhibitor exposure, one out of six patients with CRC and none of three patients with NSCLC had objective responses. Among KRAS G12C inhibitor-naïve patients, 1 of 12 patients with CRC and 3 of 15 patients with NSCLC had objective responses (35).

Avutometinib is a dual RAF and MEK inhibitor, and it is investigated as a combination therapy with sotorasib for previously treated advanced KRAS G12C-mutated advanced NSCLC in a phase Ib/II trial (RAMP203, NCT05074810). Fifteen patients were included in the trial, and the most common TRAEs were nausea, AST increase, diarrhea, fatigue, and pruritus. Most TRAEs were grade 1 or 2, but grade 3 or greater alkaline phosphatase increase, diarrhea, and pruritus were observed in 20%, 13%, and 13%, respectively. Among the patients previously treated with a KRAS G12C inhibitor, the confirmed response rate was 14.3%, while 40% of KRAS G12C inhibition-naïve patients had a confirmed response (36).

## Currently available data on combining KRAS G12C inhibitors and immunotherapy or chemotherapy

Another way to enhance the efficacy of KRAS G12C inhibition is to combine it with other agents with anti-tumor efficacy, such as immunotherapy and chemotherapy (**Table 2**).

**Table 2:** Summary of currently available data on combining KRAS G12C inhibitors with immune checkpoint inhibitors and chemotherapies.

Combination (NCT number)	Phase	Population*	N	Toxicity data	Efficacy data	Reference
Pembrolizumab + adagrasib NCT04613596 (KRYSTAL-7)	2	Untreated advanced NSCLC	148	Common TRAE: nausea, diarrhea, AST/ALT increase Grade 5 TRAE: pneumonitis and pneumonia TRAE to d/c regimen: 4% for both, 6% for adagrasib, and 11% for pembrolizumab	In PD-L1 ≥ 50% (N = 54) RR: 63% mPFS: not reached	(38)
Pembrolizumab/atezolizumab + Sotorasib (lead-in and concurrent) NCT03600883, NCT04185883 (CodeBreaK 100/101)	1b	Advanced NSCLC with no prior KRAS G12C treatment	58	G3-4 TRAE: 45% with lead-in, 72% with concurrent (most commonly AST/ALT increased) TRAE to d/c regimen: 24% with lead-in, 52% with concurrent	RR (lead-in): 31% RR (concurrent): 28% mOS: 15.7m	(39)
Pembrolizumab + MK1084 NCT05067283	1	Untreated advanced NSCLC	24	Common TRAE: increased AST/ALT, diarrhea, pruritus G3-4 TRAE: 42% (AST/ALT increase most common: 13%) TRAE to d/c regimen: 25%	RR: 71%	(40)
Carboplatin + pemetrexed + sotorasib NCT04185883 (CodeBreaK 101)	1b	Advanced NSCLC	38†	Common TRAE: neutropenia, anemia, thrombocytopenia G3-4 TRAE: 58% (most commonly neutropenia) TRAE to d/c regimen: 8% (first line) and 15% (second line) for sotorasib, 4% (first line) and 15% (second line) for carboplatin, and 12% (first line) and 23% (second line) for pemetrexed	As first line (N = 20) RR: 65% As second line (N = 13) RR: 54%	(41)
Carboplatin + pemetrexed + sotorasib jRCT2051210086 (SCARLET)	2	Untreated advanced NSCLC	30	Common TRAE: anemia, neutrophil count decreased, nausea, and platelet count decreased G3-4 TRAE: mostly hematological toxicities One treatment-related death from pneumonia	RR: 88.9% mPFS: 5.7m	(42)

d/c, discontinue; G3, 4, and 5, grades 3, 4, and 5; mOS, median overall survival (months); mPFS, median progression-free survival (months); N, number of participants; NSCLC, non-small cell lung cancer; PR, partial response; RR, response rate; SD, stable disease; TRAE, treatment-related adverse events.

*All participants should have KRAS G12C mutation.

†Twenty-five patients were in the first-line setting, and 13 patients were in the second-line setting.

### Combination with immunotherapy

Combining targeted therapy with immunotherapy has been intriguing especially in solid tumors for which both targeted and immunotherapy are effective. For example, adding pembrolizumab to trastuzumab (an anti-HER2 antibody) and chemotherapy improved treatment efficacy in HER2-positive gastric cancer with acceptable toxicity (43). On the other hand, it turned out that concurrent use of EGFR-targeted therapy and immunotherapy in advanced EGFR-mutated NSCLC renders an unacceptably high risk of pneumonitis (44). Especially for KRAS G12C-mutated solid tumors, the preclinical model suggested the synergistic efficacy of KRAS G12C inhibition and immune checkpoint inhibition (9), so the safety and efficacy of this combination have been a great scientific interest.

KRYSTAL-7 is a phase 2 trial to investigate the safety and efficacy of pembrolizumab combined concurrently with adagrasib in treatment-naïve patients with advanced NSCLC (NCT04613596). Among the 148 patients included, the most common TRAEs were nausea, diarrhea, and AST/ALT increase. Two grade 5 TRAEs happened, which were pneumonitis and pneumonia. TRAEs leading to discontinuation of adagrasib were seen in 6% of the cases. Among the patients with PD-L1 TPS ≥50% (N = 54), the confirmed ORR was 63% (38).

In CodeBreaK 100/101, either atezolizumab or pembrolizumab was combined with sotorasib in two different administration schedules (NCT04185883). In the lead-in cohort, patients were started on sotorasib first, and either 21 or 42 days later, immunotherapy was combined with sotorasib. In the concurrent cohort, sotorasib and immunotherapy were started concurrently. Fifty-eight patients with advanced KRAS G12C-mutated NSCLC without previous KRAS G12C inhibition were included. Grades 3–4 TRAEs happened in 45% in the lead-in cohort and 72% in the concurrent cohort, with increased AST/ALT being the most common. In the lead-in and concurrent cohorts, 24% and 52% of the patients discontinued treatment for TRAEs, respectively. The ORR was 31% and 28% in the lead-in and concurrent cohorts, respectively, and the mOS across all cohorts was 15.7 months (39).

In a phase I trial, pembrolizumab was combined with another KRAS G12C inhibitor, MK1084 (NCT05067283). In this trial, 24 patients with previously untreated, advanced KRAS G12C-mutated NSCLC were given pembrolizumab and MK1084. The most commonly observed TRAEs were increased AST/ALT, diarrhea, and pruritus. Of the patients, 42% experienced grades 3–4 TRAEs, and 25% had to discontinue the regimen due to TRAEs. The ORR was 71% (40).

### Combination with chemotherapy

A combination of chemotherapy and targeted therapy is used routinely in various types of cancers, including CRC (45), gastric cancer (46), head and neck cancer (47), and breast cancer (48). A recent report also demonstrated a longer PFS by combining chemotherapy with osimertinib in advanced EGFR-mutated NSLCL (49). Therefore, combining chemotherapy and KRAS G12C inhibitors has been investigated to enhance the therapeutic effect.

In a sub-cohort of CodeBreaK 101, sotorasib was combined with carboplatin and pemetrexed for patients with advanced KRAS G12C-mutated NSCLC. Among the 38 patients, the most common TRAEs were neutropenia, thrombocytopenia, and anemia. A total of 58% of the patients experienced grades 3–4 TRAEs, most commonly neutropenia. In the first-line setting, 8%, 4%, and 12% of the patients needed to discontinue sotorasib, carboplatin, and pemetrexed, respectively, due to TRAEs. In the second-line setting, TRAEs leading to discontinuing sotorasib, carboplatin, and pemetrexed were observed in 15%, 15%, and 23% of the cases, respectively. In the second-line setting (13 evaluable patients), the ORR was 54%, while the ORR was 65% in the first-line setting (20 evaluable patients) (41).

SCARLET is a phase II, single-arm trial for patients with previously untreated advanced NSLCL with KRAS G12C mutation. In the trial, patients received a combination of carboplatin, pemetrexed, and sotorasib. Thirty patients were included, and the most common TRAEs observed were anemia, neutropenia, nausea, and thrombocytopenia. Grades 3–4 TRAEs were mostly hematological toxicities, but there was one treatment-related death from pneumonia. The ORR was 88.9%, and mPFS was 5.7 months (42).

## Discussion

### Summary of current data of KRAS G12C combination

Thus far, multiple clinical trials in different phases have suggested the efficacy and safety of combining KRAS G12C inhibitors with RTK inhibitors, especially EGFR inhibitors, for advanced KRAS G12C-mutated CRC (20, 27–31). Combining trametinib with sotorasib could also have some efficacy in KRAS G12C-mutated CRC (35). Given the low efficacy of KRAS G12C monotherapies for CRC, combination therapies will likely be the main focus of investigation in KRAS G12C-mutated CRC.

For NSCLC, the combinations of KRAS G12C inhibitors with afatinib (32), SHP2 inhibitors (33, 34), MEK inhibitors (35, 36), immunotherapies (38–40), and chemotherapy (41) have been reported. Based on the currently available evidence, the synergistic efficacy of combining targeted therapy with KRAS G12C inhibitors has yet to be clearly demonstrated. The combination of KRAS G12C inhibitors and immunotherapy showed more toxicity especially that the concurrent use of pembrolizumab or atezolizumab and sotorasib showed a 72% incidence of grades 3–4 TRAEs (39). This might not be a class effect since the combination of pembrolizumab and MK1084 demonstrated a lower rate of grades 3–4 TRAEs (40). It is still unclear if adding immunotherapy to KRAS G12C inhibitors ubiquitously enhances the efficacy of KRAS G12C inhibition in advanced KRAS G12C-mutated NSCLC from currently available data.

CRC and NSCLC are the dominant types of cancer clinically investigated for the combination treatments of KRAS G12C, but clinical trials have included a minority of patients with other types of cancer. One trial reported a confirmed partial response from RMC4630 (SHP2 inhibitor) plus sotorasib in a patient with ovarian cancer (34).

### Other ongoing clinical trials of KRAS G12C inhibitor combinations and their rationale

Numerous clinical trials are ongoing to investigate the safety and efficacy of other combinations for KRAS G12C-mutated solid tumors in addition to the combinations with the targets mentioned above. These are summarized in **Table 3**.

**Table 3:** Other clinical trials of KRAS G12C inhibitor combination without available clinical data.

Target	Combination	NCT number	Phase	Population*	Current status†
RTK	Cetuximab + adagrasib (KRYSTAL-10)	NCT04793958	3	Advanced CRC progressed on the first line chemotherapy	Recruiting
	Cetuximab + JDQ443 (KontRASt-03)	NCT05358249	1/2	Advanced solid tumors	Recruiting
	Cetuximab + JAB21822	NCT05002270	1/2	Advanced solid tumors	Recruiting
	Cetuximab + IBI351	NCT05497336	1	Metastatic CRC	Recruiting
	Cetuximab + adagrasib + irinotecan	NCT05722327	1	Advanced CRC	Not yet recruiting
	Cetuximab/erlotinib + LY3537982 (LOXO-RAS-20001)	NCT04956640	1	Advanced solid tumors	Recruiting
	Erlotinib + divarasib	NCT04449874	1	Advanced solid tumors	Recruiting
	Panitumumab + sotorasib (ComboMATCH)	NCT05638295	2	Advanced solid tumors	Not yet recruiting
	Tarloxotinib + sotorasib	NCT05313009	1	Advanced NSCLC progressed on KRAS G12C inhibitors	Active, not recruiting
SHP2	TNO155 + sotorasib (CodeBreaK 101)	NCT04185883	1/2	Advanced solid tumors	Recruiting
	TNO155 + adagrasib (KRYSTAL-2)	NCT04330664	1/2	Advanced solid tumors	Active, not recruiting
	TNO155 + LY3537982 (LOXO-RAS-20001)	NCT04956640	1	Advanced solid tumors	Recruiting
	TNO155 + tislelizumab + JDQ443	NCT04699188	1/2	Advanced solid tumors	Recruiting
	BBP398 + sotorasib	NCT05480865	1	Advanced solid tumors	Recruiting
	GDC1971 + divarasib	NCT04449874	1	Advanced solid tumors	Recruiting
	JAB3312 + JAB21822	NCT05288205	1/2	Advanced solid tumors	Recruiting
	SAR442720 + adagrasib	NCT04418661	1/2	Advanced solid tumors	Active, not recruiting
	RMC4630 + sotorasib	NCT05054725	2	Advanced NSCLC progressed on prior standard therapies	Active, not recruiting
SOS1	BI1701963 + adagrasib (KRYSTAL-14)	NCT04975256	1	Advanced solid tumors	Completed
	BI1701963 + sotorasib (CodeBreaK 101)	NCT04185883	1/2	Advanced NSCLC or CRC	Recruiting
	BI1701963 + BI-1823911	NCT04973163	1	Advanced solid tumors	Active, not recruiting
	MRTX0902 + adagrasib	NCT05578092	1/2	Advanced solid tumors	Recruiting
Wild-type RAS	RMC6236 + RMC6291	NCT06128551	1	Advanced solid tumors	Recruiting
MEK	Avutometinib + adagrasib (RAMP 204)	NCT05375994	1/2	Advanced NSCLC progressed on KRAS G12C inhibitors	Recruiting
	Trametinib + JDQ443 (KontRASt-03)	NCT05358249	1/2	Advanced solid tumors	Recruiting
ERK	Temuterkib + LY3537982 (LOXO-RAS-20001)	NCT04956640	1	Advanced solid tumors	Recruiting
	ERAS-007 + sotorasib (HERKULES-2)	NCT04959981	1	Advanced NSCLC	Completed
PI3K	Inavolisib + divarasib	NCT04449874	1	Advanced solid tumors	Recruiting
mTOR	Everolimus + sotorasib (CodeBreaK 101)	NCT04185883	1/2	Advanced solid tumors	Recruiting
	Nab-sirolimus + adagrasib (KRYSTAL-19)	NCT05840510	1/2	Advanced solid tumors	Recruiting
CDK4/6	Palbociclib + adagrasib (KRYSTAL-16)	NCT05178888	1	Advanced solid tumors	Active, not recruiting
	Palbociclib + sotorasib (CodeBreaK 101)	NCT04185883	1/2	Advanced solid tumors	Recruiting
	Abemaciclib + LY3537982	NCT04956640	1	Advanced solid tumors	Recruiting
	Ribociclib + JDQ443 (KontRASt-03)	NCT05358249	1/2	Advanced solid tumors	Recruiting
AURKA	LY3295668 + LY3537982 (LOXO-RAS-20001)	NCT04956640	1	Advanced solid tumors	Recruiting
ULK1/2	DCC-3116 + sotorasib	NCT04892017	1/2	Advanced NSCLC	Recruiting
PARP	Olaparib + adagrasib	NCT06130254	1	Advanced solid tumors	Not yet recruiting
CXCR1/2	Ladarixin + adagrasib	NCT05815173	1	Advanced NSCLC	Recruiting
VEGF	Bevacizumab-awwb + FOLFIRI/FOLFOX + sotorasib (CodeBreaK 101)	NCT04185883	1/2	Advanced CRC	Recruiting
	Bevacizumab + divarasib	NCT04449874	1	Advanced solid tumors	Recruiting
PD-1	Pembrolizumab + adagrasib	NCT04418661	1/2	Advanced solid tumors	Active, not recruiting
	Pembrolizumab + adagrasib (KRYSTAL-17)	NCT05609578	2	Untreated advanced NSCLC with TPS ≥1% and TPS <50%	Recruiting
	Pembrolizumab + divarasib	NCT05789082	1/2	Untreated advanced NSCLC	Recruiting
	Pembrolizumab + LY3537982 (LOXO-RAS-20001)	NCT04956640	1	Advanced solid tumors	Recruiting
	Pembrolizumab + RMC6291	NCT06162221	1/2	Advanced solid tumors	Recruiting
	Nivolumab + adagrasib (as neoadjuvant treatment, NeoKAN)	NCT05472623	2	Resectable NSCLC	Recruiting
PD-L1	Durvalumab + adagrasib	NCT05848843	1	Advanced NSCLC and GI cancers	Not yet recruiting
	Atezolizumab + divarasib	NCT04449874	1	Advanced NSCLC	Recruiting
	AMG404 + sotorasib (CodeBreaK 101)	NCT04185883	1/2	Advanced solid tumors	Recruiting
	Sintilimab ± chemotherapy + IBI351	NCT05504278	1	Advanced NSCLC	Recruiting
	Tislelizumab ± TNO155 + JDQ443 (KontRASt-01)	NCT04699188	1/2	Advanced solid tumors	Recruiting
Chemotherapy	Carboplatin/cisplatin + pemetrexed + sotorasib	NCT05118854	2	Resectable non-squamous NSCLC	Recruiting
	Platinum doublet chemotherapy + sotorasib (vs. pembrolizumab) (CodeBreaK 202)	NCT05920356	3	Untreated advanced NSCLC	Recruiting

BTC, biliary tract cancer; CRC, colorectal cancer; IC, immune checkpoint; GI, gastrointestinal; NSCLC, non-small cell lung cancer; PDAC, pancreatic ductal adenocarcinoma.

*All participants should have KRAS G12C mutation.

†Based on the registered status as of 12/1/2023 at ClinicalTrials.gov.

#### SOS1

**Figure 1:**
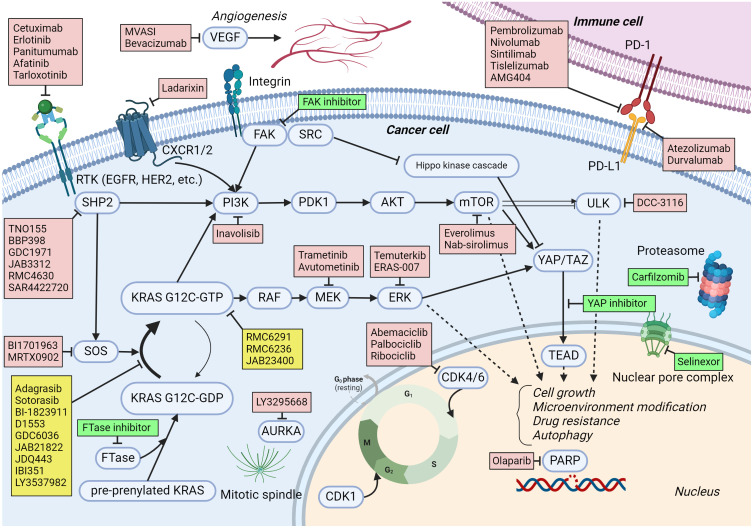
KRAS G12C signaling pathways and medications under trial in combination with KRAS G12C inhibitors. The activating and inhibiting signaling are expressed by pointed head and blunt head arrows, respectively. Arrows with broken lines mean a multi-step effect rather than a direct effect. Medications to target KRAS G12C are listed in yellow boxes. Medications in red boxes are being investigated in clinical trials as combination treatments with KRAS G12C inhibitors. Medications in green boxes are not currently in clinical trials but have the potential to be combined with KRAS G12C inhibitors based on preclinical evidence. Created with BioRender.com.

SOS1 is a guanine nucleotide exchange factor that controls the activation of the KRAS G12C protein through its nucleotide-exchanging function (50) (**Figure 1**). Preclinical evidence suggests that the inhibition of SOS1 attenuates feedback reactivation induced by MEK inhibitors in KRAS-dependent cancers improving sensitivity to MEK inhibitors (51). Another in vitro study indicated that the combination of SOS1 inhibitors and MEK inhibitors could overcome acquired resistance from secondary mutations to KRAS G12C inhibitors in NSCLC (52). Both feedback activation of collateral pathways and secondary mutations are important resistance mechanisms to KRAS G12C inhibitors, so multiple clinical trials have been conducted to combine SOS1 and KRAS G12C inhibitors (NCT04975256, NCT04185883, NCT04973163, NCT05578092).

#### Wild-type RAS

The feedback activation of wild-type RAS, such as HRAS and NRAS, is an important resistance mechanism to KRAS G12C inhibition (53). RMC6236 is another oral RAS (ON) inhibitor. The inhibitory complex with cyclophilin A binds GTP-bound RAS protein with or without mutations. A phase I study including patients with solid tumors harboring any missense mutation at KRAS G12 showed promising anti-tumor activity with reasonable safety (54). There is a phase I trial that combines RMC6236 with RMC6291, a KRAS G12C (ON) inhibitor, for patients with KRAS G12C-mutated advanced solid tumors (NCT06128551).

#### ERK

ERK is a downstream component of the RAS–RAF–MEK–ERK pathway and plays a role in cell cycle regulations (**Figure 1**). An in vitro study demonstrated that treatment with KRAS G12C inhibitors for several cell lines, such as H358, leads to an initial suppression of ERK within 24 h followed by a rebound activation of ERK (55). This could be a mechanism of the primary resistance to KRAS G12C inhibitors. Therefore, several early-phase clinical trials have been conducted to combine ERK inhibitors with KRAS G12C inhibitors (NCT04956640; Temuterkib + LY3537982, NCT04959981; ERAS-007 + Sotorasib).

#### PI3K

PI3K is a component of the PI3K–AKT–mTOR pathway and regulates cell growth, proliferation, differentiation, and motility. The activity of PI3K is regulated by many signaling molecules, such as SHP2 and FAK (**Figure 1**). Due to its important role in cancer development and progression, the inhibition of PI3K has been investigated, and several PI3K inhibitors have been approved as cancer treatments (56). Since the PI3K–AKT–mTOR pathway could bypass the RAS–RAF–MEK–ERK pathway, it may contribute as a collateral pathway for KRAS G12C inhibition resistance (23). It was reported that in cell lines resistant to sotorasib, PI3K–AKT was constitutively activated (57). An in vivo study demonstrated a synergistic effect of PI3K inhibitors with KRAS G12C inhibitors in an NSCLC model (58). An in vitro study also demonstrated a synergistic effect of PI3K inhibitors with KRAS G12C inhibitors in PDAC cell lines (59). A phase I trial to investigate inavolisib (PI3K inhibitor) combined with divarasib is ongoing (NCT04449874).

#### mTOR

mTOR is a protein kinase that regulates cell cycle, proliferation, and motility. Multiple molecules interfere with and regulate mTOR, including PI3K and AKT, which are important upstream regulators in cancer (**Figure 1**). Several mTOR inhibitors have been approved to treat solid tumors (60). Dysregulation of mTOR could be a collateral pathway that causes resistance to KRAS G12C inhibitors (61). In vivo and in vitro studies demonstrated the enhanced anti-tumor activity from combining mTOR inhibitors, such as everolimus, with KRAS G12C inhibitors (61–63). There are early-phase clinical trials combining mTOR inhibitors (everolimus and nab-sirolimus) with KRAS G12C inhibitors (NCT04185883, NCT05840510).

#### CDK4/6

CDK4/6 are protein kinases that regulate the cell cycle and contribute to cell growth and proliferation (**Figure 1**). CDK4/6 is regulated by D-type cyclins and CDK inhibitor p16. CDK4/6 inhibitors are effective as a treatment for breast cancer (64). The activation of CDK4/6 plays a role in KRAS G12C inhibitor resistance through co-alteration of CDKN2A and increased activation from collateral pathways such as the PI3K–AKT–mTOR pathway (24, 61). Multiple in vitro and in vivo studies demonstrated enhanced activity of KRAS G12C inhibitors combined with CDK4/6 inhibitors (62, 65). Clinical trials to combine CDK4/6 and KRAS G12C inhibitors are ongoing (NCT05178888, NCT04185883, NCT04956640, NCT05358249).

#### AURKA

Aurora kinase A (AURKA) is a protein kinase that plays a critical role in properly forming the mitotic spindle, an essential process for mitosis (**Figure 1**). An in vitro study of KRAS G12C-mutated NSCLC cells revealed that AURKA is upregulated in the tumors developing resistance to KRAS G12C inhibition. Individual knockout of AURKA augmented the efficacy of KRAS G12C inhibition, and AURKA inhibitors suppressed the reactivation of KRAS on G12C inhibition (66). A phase I clinical trial is ongoing to investigate the combination of LY3295668 (an AURKA inhibitor) and LY3537982 (a KRAS G12C inhibitor) for advanced KRAS G12C-mutated solid tumors (NCT04956640).

#### ULK1/2

ULK1/2 is a kinase involved in autophagy with numerous downstream phosphorylation targets. ULK1/2 has a complex interplay with mTORC1 and AMPK (5-AMP-activated protein kinase) (67) (**Figure 1**). There is no FDA-approved ULK inhibitor as of yet. An in vitro study showed increased ULK1 activity and autophagy in KRAS G12C-mutated NSCLC cell lines when treated with KRAS G12C inhibitors, which was suppressed by a ULK1/2 inhibitor. Both in cell line- and patient-derived xenograft models, combining a ULK1/2 inhibitor and a KRAS G12C inhibitor rendered higher efficacy in tumor control (68). A combination of DCC-3116 (a ULK1/2 inhibitor) and sotorasib for KRAS G12C-mutated NSCLC is being investigated clinically (NCT04892017).

#### PARP

PARP detects and initiates cellular response to single-strand DNA breaks (**Figure 1**). There are multiple PARP inhibitors approved for multiple types of cancer, including ovarian, breast, pancreatic, and prostate cancer, especially with homologous recombination repair gene mutations (69). Sun et al. demonstrated a synergistic efficacy of combining MEK and PARP inhibitors for RAS-mutated cancers in vitro and in vivo (70). In their study, the activated RAS/MAPK pathway suppressed FOXO3a expression, which was associated with resistance to PARP inhibitors. MEK inhibitors could upregulate FOXO3a expression and sensitize RAS-mutated cancer to PARP inhibitors. Although there is no publicly available preclinical evidence, it might be extrapolated from this finding that KRAS G12C inhibitors may sensitize KRAS G12C-mutated cancers to PARP inhibitors by suppressing the RAS/MAPK pathway and increasing FOXO3a expression. A phase I clinical trial combines olaparib and adagrasib for advanced solid tumors (NCT06130254).

#### CXCR1/2

CXCR1/2 is expressed on leukocytes and cancer cells. By interfering with CXCL8, CXCR1/2 activates variable signaling pathways in cancer cells, including the RAS–RAF–MEK–ERK and PI3K–AKT–mTOR pathways (**Figure 1**). There is no approved CXCR1/2 inhibitor, but several agents are under investigation (71). Ladarixin is one of the CXCR1/2 inhibitors, and in combination with a PD-1 inhibitor, it showed effectiveness in cell line- and patient-derived xenograft models of PDAC (72). A phase I clinical trial combining ladarixin and adagrasib for advanced NSCLC is currently ongoing (NCT05815173).

#### VEGF

VEGF is a protein produced by many types of cells, including cancer cells, and it enhances angiogenesis (**Figure 1**). Inhibiting the signaling from VEGF and VEGFR interaction by monoclonal antibodies (bevacizumab or ramucirumab) or small molecules (sorafenib, lenvatinib, cabozantinib) has shown effectiveness in various types of cancer. Since the previous clinical trial for CRC showed additional benefit from bevacizumab to chemotherapy regimen (73), bevacizumab is combined with KRAS G12C inhibitors in early-phase clinical trials (NCT04185883, NCT04449874).

### Other possible therapeutic targets combined with KRAS G12C inhibition and rationale

Other potential oncogenic targets can be co-inhibited with KRAS G12C for which a clinical trial has not yet been initiated. We herein review these possible therapeutic targets and their preclinical rationale.

#### YAP/TAZ-TEAD

YAP is a transcription coregulator involved in cell proliferation and apoptosis suppression. TAZ is a YAP paralog and plays a role in cell proliferation. YAP/TAZ enters the nucleus in an active state to interact with TEAD leading to enhanced transcription (**Figure 1**). YAP/TAZ is negatively regulated by the Hippo signaling pathway comprising various molecules, including MST 1/2 and LATS 1/2 (74). Dysregulation of YAP-/TAZ-mediated transcriptional activity has been observed in types of cancer, and the inhibition of YAP–/TAZ–TEAD interaction has been a promising treatment target for cancer. A small molecule inhibitor that blocks the interactions between YAP/TAZ and TEAD (GNE-7883) showed anti-tumor activity for various types of cancer in vitro and in vivo (75). Furthermore, both primary and acquired resistance to KRAS G12C inhibitors were reversed by GNE-7883 by inhibiting YAP–/TAZ–TEAD interaction in vivo (75). Thus, co-inhibition of YAP–/TAZ–TEAD and KRAS G12C could be a promising combination for KRAS G12C-mutated advanced cancers when the safety and efficacy of YAP–/TAZ–TEAD inhibitors are established in ongoing early-phase clinical trials (NCT05228015, NCT04665206).

#### FAK

FAK is a non-receptor tyrosine kinase and an adaptor protein that regulates adhesion signaling and cell migrations, but it also contributes to cell survival in malignancy (76). FAK is activated by integrin or RAS homolog family member A signaling. FAK activates numerous cell signaling pathways, including the RAS–RAF–MEK–ERK and PI3K–AKT–mTOR pathways. It also regulates the Hippo kinase cascade, activating YAP–/TAZ–TAD interaction (77, 78) (**Figure 1**). Despite the well-established preclinical evidence, single-agent FAK inhibitors have shown limited efficacy in early-phase clinical trials (79, 80). Alternatively, FAK inhibitors are currently investigated in combination with immunotherapies (NCT03727880), chemotherapies (NCT03287271), and targeted therapies (NCT04620330). An in vitro and in vivo study showed that sustained activation of FAK in KRAS G12C inhibition led to KRAS G12C resistance, and the combination of a FAK inhibitor and a KRAS G12C inhibitor showed a synergistic anti-tumor effect in KRAS G12C-mutated xenografts (81). Besides the combinations currently under investigation, the FAK and KRAS G12C inhibition combination could be considered for a clinical trial.

#### Farnesyltransferase

Farnesyltransferase (FTase) is one of the prenyltransferases and modifies proteins by adding isoprenoid lipids posttranslationally. FTase targets the RAS family, including KRAS, NRAS, and HRAS, as well as Ras homolog enriched in brain (RHEB). Prenylation by FTase activates RAS and RHEB proteins through proper membrane localization leading to cell proliferation through the RAS–RAF–MEK–ERK and PI3K–AKT–mTOR pathways (82, 83). (**Figure 1**). Since FTase contributes to oncogenesis by interacting with RAS and RHEB genes, FTase inhibitors have been investigated as an anti-tumor treatment (84). Tipifarnib is an FTase inhibitor investigated in early-phase clinical trials (85, 86). Activation of mTOR contributes to developing the resistance to KRAS G12C inhibitors, so suppressing mTOR with RHEB inhibition through FTase inhibitors could enhance the efficacy of KRAS G12C inhibitors. In KRAS G12C mutant NSCLC cell lines and patient-derived xenograft models, the combination of an FTase inhibitor (KO-2806) and adagrasib demonstrated markedly enhanced antitumor effects (87). The cells that co-inhibited with KO-2806 and adagrasib showed reduced ERK phosphorylation and mTOR signaling. Although tipifarnib has shown an unfavorable toxicity profile, especially regarding bone marrow suppression when combined with cytotoxic chemotherapy, it was better tolerated as a monotherapy, though its efficacy was more limited (88, 89). Since KRAS G12C inhibitors are not very myelosuppressive, combining KRAS G12C and FTase inhibitors might have reasonable safety and efficacy. Additionally, KO-2806 is a next-generation FTase inhibitor in an ongoing phase 1 clinical trial, which may demonstrate a better safety profile (NCT06026410).

#### Proteasome

Proteasome degrades most cellular proteins under strict control, contributing to proper cell cycle, transcription, signaling, trafficking, and protein quality control. The dysregulation of the proteasome is closely associated with various diseases, including malignancy (90). The FDA has approved multiple proteasome inhibitors as a treatment for multiple myeloma but not for solid tumors yet. Carfilzomib is one of the FDA-approved proteasome inhibitors and was reported to suppress integrin B4 (ITGB4) expression (91). Mohanty et al. have demonstrated that increased expression of ITGB4 and activation of the WNT/β-catenin signaling pathway contribute to sotorasib resistance in KRAS G12C-mutated cell lines. Their in vitro and in vivo study demonstrated a synergistic anti-tumor efficacy from the combination of carfilzomib and sotorasib in NSCLC cell lines and xenografts through downregulating ITGB4 and β-catenin expression (92).

#### Nuclear transport

XPO1 mediates the exportation of various proteins and RNAs from the nucleus (**Figure 1**). Dysregulated nuclear export is observed in several types of cancer (93). Selinexor is a selective inhibitor of XPO1 inhibitor and has been approved for multiple myeloma and diffuse large B-cell lymphoma. Previously, a general dependency of KRAS-mutated NSCLC on XPO1 was reported, and Selinexor monotherapy reduced tumor growth in multiple patient-derived lung adenocarcinoma xenografts (94). The combination of Selinexor and sotorasib has also shown enhanced anti-tumor activity through downregulating cell cycle marker expression and increased nuclear accumulation of tumor-suppressor proteins in vitro and in vivo (95).

### Beyond KRAS G12C inhibition

Although KRAS G12C inhibitions and their combination therapies have shown efficacy, the proportion of patients benefitting from KRAS G12C inhibition is limited since G12C mutation is just a minority of KRAS alterations. Other treatment strategies for KRAS-altered solid tumors are investigated, including KRAS G12D and pan-RAS inhibition.

Multiple KRAS G12D inhibitors are currently under investigation, and MRTX1133 is one of them. MRTX1133 is a selective and non-covalent KRAS G12D inhibitor and has shown anti-tumor efficacy in vivo and in vitro models, especially in KRAS G12D mutant PDAC models (96). A phase I/II clinical trial of MRTX1133 for KRAS G12D-mutated solid malignancy is ongoing (NCT05737706). Multiple early-phase clinical trials of other KRAS G12D inhibitors, including RMC9805 (NCT06040541) and HRS4642 (NCT05533463), are also ongoing. While these therapeutics inhibit KRAS G12D by locking the molecule in an inactive state (OFF inhibitor) or prohibiting active molecules from activating downstream signaling (ON inhibitor), a brand new approach for KRAS inhibition, protein degradation, is being developed. ASP3082 is a protein degrader that connects mutated KRAS G12D with E3 ligase to initiate the degradation process through ubiquitination. An in vivo study demonstrated its anti-tumor efficacy in PDAC, CRC, and NSCLC models (97). A phase I trial of ASP3082 is currently ongoing (NCT05382559).

More therapeutics targeting a wider range of KRAS mutations are under investigation, including RMC6236. Another pan-KRAS inhibitor that blocks nucleotide exchange to prevent the activation of wild-type KRAS and a broad range of KRAS mutations showed tumor-suppressive effects in vivo (98).

## Conclusion

We herein reviewed the latest evidence of KRAS G12C inhibitor combination therapies. Although none of the KRAS G12C inhibitor combinations has been approved for clinical use, available data suggest the safety and efficacy of some combinations. Numerous ongoing clinical trials combine KRAS G12C inhibitors with other therapeutics that target upstream, downstream, or collateral pathways of the RAS–RAF–MEK–ERK pathway, as well as chemotherapy, anti-VEGF, and immunotherapies. Preclinical evidence suggests other possible targets that have not yet entered into clinical investigation. The area of investigation is now expanding to non-G12C mutations of KRAS, and preclinical and early clinical evidence has demonstrated promising results. Further development of potent KRAS inhibitors, as well as rational combination with other therapeutics, are likely to change the treatment landscape for KRAS-mutated malignancies.

## Author contributions

HM: Writing – original draft. SK: Writing – review & editing. DH: Writing – review & editing.
